# A Novel Defect Tolerance Scheme for Nanocrossbar Architectures with Enhanced Efficiency

**DOI:** 10.3390/mi10010014

**Published:** 2018-12-27

**Authors:** Devisree Sasikumar, Anand Kumar

**Affiliations:** Electrical and Electronics Engineering, BITS Pilani, Dubai Campus, Dubai 345055, United Arab Emirates; akumar@dubai.bits-pilani.ac.in

**Keywords:** molecular switches, nanocrossbar architecture, faults, stuck-at-off, stuck-at-on, fault tolerance, maximum independent set, yield

## Abstract

The semiconductor industry is now facing challenges to keep pace with Moore’s law and this leads to the requirement of new materials and newer technological devices. Molecular switch-based nanodevices are one of the promising areas because of their ultimate size and miniaturisation potential. These nanodevices are built through a self-assembled bottom-up manufacturing method in which the possibility of external intervention is negligible. This leads to a considerable yield loss due to defective device production and the traditional test-and-throw faulty device approach will not hold well. Design of fault-tolerant devices are the only possible solution. A widely studied nanodevice is nanocrossbar architectures and their fault tolerance can be designed by exploiting the programmable logic array’s fault tolerance schemes. A defect-unaware fault tolerance scheme is developed in this work based on the bipartite graph analogy of crossbar architectures. The newly-designed algorithm can eliminate more than one node in each iteration and, hence, a defect-free subcrossbar can be obtained much faster compared to the existing algorithms. A comparison with the existing defect-unaware fault-tolerant methods with this newly-developed algorithm shows a better yield in most of the cases.

## 1. Introduction

With increased counts, transistors were able to operate at higher frequency until recently, and the “performance at any cost” tactic will soon lead to fundamental thermal limits of an integrated circuit (IC), approaching its end [1]. This has led to the implementation of new materials and architectures. Carbon nanotubes (CNT), semiconductor nanowires, molecular devices, spin transistors, single-electron transistors. etc., are the emerging nanotechnology devices. Most of these nano electronic devices possess a regular array-like structure generated by a stochastic bottom-up assembly. In this method, individual components are built first and then the design is assembled onto it. By utilizing specific intra-molecular interactions, regular array-like structures are easy to produce with this kind of manufacturing technology [2]. The nanocrossbar (NC) architecture is a promising technology to improve the computing performance of nano electronic devices. The NC structure with a bistable molecular switch as the switching element is known as a molecular switch crossbar circuit and the most important advantage is their smallest size (molecular dimension). In an NC architecture, the molecular switches are connected to each other in a two-dimensional grid. Since two terminal devices, like molecular switches in crossbar circuits, are more advantageous than three terminal devices in terms of flexible designs and reduced area requirement, they become the dominant category device [3].

During the fabrication life cycle of an electronic component, it is impossible to achieve 100% efficiency and, hence, it will lead to faulty IC production. Since the manufacturing of CMOS devices is through a controlled top-down approach, defect density is low, hence, it is cost effective to reject an IC if found defective during the testing process. The nanodevices are more susceptible to defects because of their ‘self-assembled bottom-up’ manufacturing approach [2]. This will lead to a considerable yield loss and, hence, the test-and-throw faulty device approach will not hold well [4]. Thus, the only available option in the case of nanodevices for high throughput are the use of fault tolerance methods. 

A nanocrossbar architecture-based electronic device can have errors either on the nanowire or in the switching element. Faults in a nanocrossbar structure can be broadly classified into two categories; (1) permanent faults or defects, and (2) transient faults [5]. Defects that occur during the fabrication process are permanent faults, and in the case of molecular switching devices these may occur as a structural differences in the molecule. Transient faults occur during the operation of the device and, hence, they are considered as field errors. Nanocrossbar architectures are very similar to programmable logic arrays (PLA) in terms of programming features, structure, and utilization [6]. In 1990, Demjanenko and Upadhyaya developed a fault-tolerant scheme for PLAs on the basis of a bipartite graph model. Hence, bipartite graph-based fault-tolerant schemes will be suitable for designing a fault-tolerant nanocrossbar architecture irrespective of the type of switching element. A disadvantage of the bipartite graph-based fault-tolerant scheme is that these methods are not suitable for wire defects and transient faults.

## 2. Nanocrossbar Architecture as a Bipartite Graph

**Figure 1:**
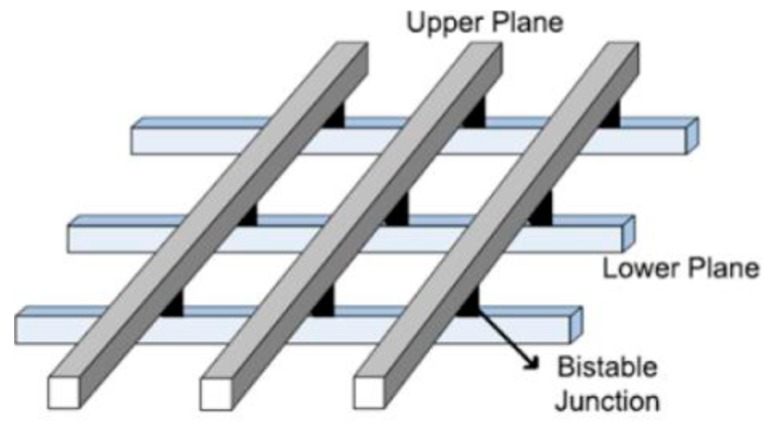
Schematic representation of a nanocrossbar (NC) architecture.

Nanocrossbar architectures are realized by using molecular switch or any other two terminal nanodevice as the programming element. These switching elements are connected to each other in a two-dimensional grid. A schematic representation of an NC architecture is given Figure 1 [7].

A layer of bistable [2] rotaxane-like molecular switch is sandwiched between the metal nanowires to form molecular crosspoint devices. These devices will act as an electrically-toggled switch for implementing the logic function. These nanowires are aligned parallel to each other in two different planes and these planes are placed orthogonally. Nanoimprint lithography is a technique that can produce devices even at the sub-10 nm feature size.

### 2.1. Defects in a Crossbar Architecture

Nanocrossbar architectures thus manufactured will have a very high fault rate because of their self-assembly. A nanocrossbar architecture-based electronic device can have errors either on the nanowire or in the switching element. In this work, only switching element defects are considered and nanowires are assumed to be defect-free. The most common type of faults occurring in nanocrossbar circuits are due to defects and they are permanent faults. Molecular switching elements may lose their switching capability during the manufacturing process and such switches are considered as a defective switch. Defects in a molecular switching element can be of three categories: (1) stuck-at-off, (2) stuck-at-on [8], and (3) neither stuck-at-off nor stuck-at-on [9].

- ***Stuck-at-off***: These type of defects makes the corresponding crosspoint switch not capable of conducting electrical current or, in the case of a molecular switch, the molecule can possess only its ground state co-conformer (GSCC) geometry.
- ***Stuck-at-on fault***: These defects make the corresponding switch constantly conduct electric current or, in case of a molecular switch, their geometry is permanently stuck in the metastable state co-conformer (MSCC) state.
- ***Neither stuck-at-off nor stuck-at-on fault***: This type of fault is typically found in molecular switching elements. Here, the molecule loses its intended switching action, but it is neither stuck-at-off nor stuck-at-on position.
- Our previous studies presented a detailed analysis on these type of faults [9] and in this paper we are concentrating only on the fault tolerance method for stuck-at faults.

### 2.2. Characterisations of a Bipartite Graph

- *Bipartite Graph*: A graph *G* is called *bipartite* if its vertex set *V* can be decomposed into two disjoint subsets *V*_1_ and *V*_2_ such that every edge in *G* joins a vertex in *V*_1_ a vertex in *V*_2_ [10]. In a bipartite graph no edge will exist between the two nodes of the same set. *Adjacent nodes* are the connected nodes via edges. *Degree* of a node is the number of edges connected to the node.
- *Bipartite complement graph*: This is the complement or inverse of a bipartite graph by keeping all of the nodes in the original graph with an addition of all edges that do not exist in the original graph. Two distinct vertices in the complement graph are adjacent if and only if they are not adjacent in the original graph [10].
- *Independent set:* This is a subset of the set of vertices which contains nodes such that no node pair has an edge connecting the nodes. Independent sets are also known as stable sets [10,11].
- *Biclique:* A biclique is complete bipartite graph. This is a special kind of graph in which every vertex of the first set of vertices is connected to every vertex of the second set of vertices [11].

### 2.3. Analogy between Crossbar Arrays and Bipartite Graphs

A nanocrossbar architecture with n input and n output wires can be represented as an *n × n* graph with nodes and edges. A crossbar architecture is generally represented as a bipartite graph. Here, each of the nodes corresponds to input and output lines. The edges connecting between these nodes represents the cross-point switch present between the particular input and output wire. The two subsets of the vertex set of the bipartite graph are represented by I and O which corresponds to the input set and the output set of the crossbar array. Each configurable cross-point switch is represented by an edge connecting between the node set I and O [11].

- Stuck-at ON fault—Erasure of the corresponding nodes from V and U along with all edges connected to these nodes.
- Stuck-at OFF fault—Erasure of the corresponding edge only.

The manufacturing yield of crossbar arrays can be maximized by implementing fault-tolerant methods. Fault-tolerant logic mapping problem is further divided into two categories: (1) defect-aware fault tolerance and (2) defect-unaware fault tolerance. All defect-tolerant designs either avoid or exploit defects. In defect avoiding approach, faulty wires and switches are avoided and defect-free crossbar subset is searched for mapping logic functions [12]. Both procedures use a defect map to show the position of defects/faults in the crossbar architecture. Hence, these methods are also called as defect avoiding and defect employing methods, respectively.

In defect-unaware methods a *k × k* defect-free subcrossbar is obtained from an *n × n* crossbar and thus size of the required crossbar is known in advance. A defect-free subcrossbar can be represented as a biclique. According to the fundamentals of graph theory, the maximum independent set of the complement graph will be the biclique of the original graph [10]. This principle is exploited in developing the new fault-tolerant method.

Logic implementation on to a defect-free subcrossbar is a direct and straight-forward mapping, but the existing literature shows that the efficiency of defect-unaware methods is significantly low and, hence, the studies are limited. In defect-aware logic mapping methods, defective elements are utilized during the mapping process and, hence, a better area yield is obtained. However, these methods are complicated to implement. The problem formalizing is comparatively easier and flexible. This leads to a very large number of studies in the defect-aware logic mapping field. 

### 2.4. Obtaining the Bipartite Graph Corresponding to a Defective Crossbar Architecture

**Figure 2:**
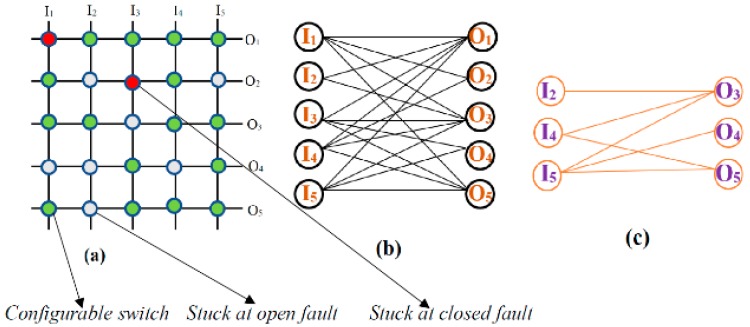
Representation of (**a**) a defective crossbar; (**b**) a bipartite graph representation of the crossbar by considering only the stuck at open fault; and (**c**) a bipartite graph representation of the crossbar by considering both stuck-at-open and -closed faults.

Consider a 5 × 5 crossbar structure with two stuck-closed faults and seven stuck-open faults, as shown in Figure 2a. Figure 2b shows the bipartite graph representation of this defective crossbar by considering the effect of stuck-open faults alone (the stuck-closed fault switch shown in Figure 2a is assumed to be fault-free in this scenario). Here, few edges are absent which corresponds to the stuck-open faults and it is to be noted that none of the nodes are absent [13]. This is because the effect of a stuck-open fault will not eliminate the complete input and/or output wire. Figure 2c shows the bipartite graph corresponding to the crossbar under consideration after looking into the effect of both stuck-open and stuck-closed faults. 

Here we can see that the some of the nodes are also eliminated from the bipartite graph representation, which indicates the presence of a stuck-closed fault which eliminates the corresponding input and output lines along with the switch [14].

## 3. A Novel Defect-Tolerant Scheme

**Figure 3:**
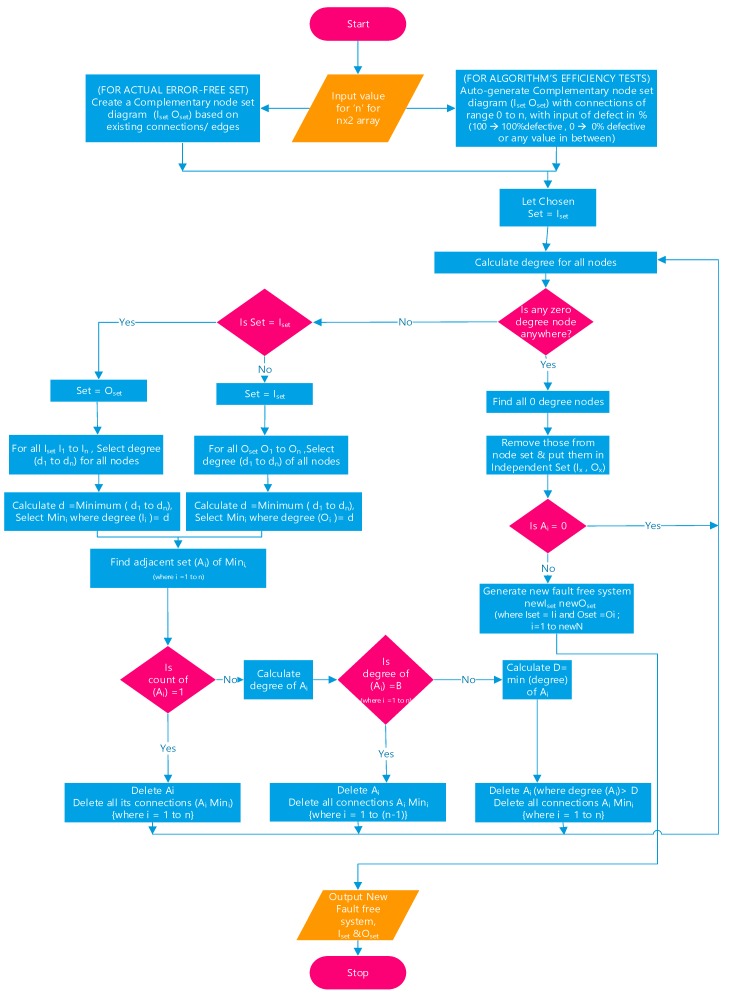
Flowchart of the novel defect-tolerant scheme.

In this newly-developed algorithm, the maximum independent set of the complement graph is calculated in a much faster and efficient manner compared to the existing [13,15,16,17] defect-unaware methods. The newly-developed defect-tolerant method’s flowchart is given in Figure 3.

The newly-developed algorithm is applied to the systems which contain stuck-open faults alone and a system that contains both stuck-open and stuck-closed faults. The algorithm finds the maximum independent set of the given bipartite complement graph within a fewer number of iterations in comparison with Yamani’s algorithm [15]. This algorithm progress as follows, resulting in the generation of the maximum independent set of the complement graph, which is nothing but the defect-free subcrossbar. Initially, the algorithm checks for all zero-degree nodes and all such nodes are moved into the independent node set. In the next step the algorithm selects all minimum degree nodes from the input node set and the adjacent nodes set is completed by considering the adjacent nodes for all the minimum degree nodes. The next phase brings the main difference in running time compared to the existing algorithms.

In this proposed algorithm, all adjacent nodes are eliminated except the minimum degree node. This situation may end up in three different scenarios: (a) If only adjacent node exists then it is removed from the node set and correspondingly its edges, too; (b) if there exist more than one adjacent node and each of them have different degrees, then all nodes except the minimum degree adjacent node/nodes are removed from the original node list; and (c) if there exist more than one adjacent node and each of them have same node degree, then any one minimum degree adjacent node is retained, and all others are eliminated. Deletion of a node explicitly indicates the deletion of the corresponding edge. As the next step, the algorithm checks the possibility of new zero-degree nodes and they are moved into the independent node set. The complete algorithm is repeated with alternating input and output nodes in each iteration, until any node set becomes empty.

The algorithm is implemented using JAVA (version 1.8. Mac OS 10.13) as the development platform used with Eclipse 4.6 as the development tool. The pseudo code of the algorithm is as given below.


**Algorithm 1. Defect tolerance algorithm with enhanced efficiency.**

 *1.*
*While Connection Exists Between Nodes*
 *2.*
*Parse Input Graph Left -> Right*
 *3.*
*Get Candidate Nodes(Graph) <- Minimum Connection Nodes(Graph)*
 *4.*
*Get Adjacent Nodes(Graph) <- Candidate Nodes(Graph)*
 *5.*
*Get Minimum(Adjacent Nodes)*
 *6.*
*If Count(#5) == 1 And Count(#4) == 1 Then Keep(#5) And Delete(All Other #4’s)*
 *7.*
*If Count(#5) == 1 And Count(#4) > 1 Then Keep(Any One #4’s) And Delete(Other #4’s)*
 *8.*
*If Count(#5) == Count(#4) Then Keep(Any One #4’s) And Delete(Other #4’s)*
 *9.*
*If Count(#5) < Count(#4) Then Keep(#5’s) And Delete(#4’s)*
 *10.*
*Get Independent Nodes(Graph)*
 *11.*
*Redraw(Graph)*
 *12.*
*Repeat #2 Right <-> Left Until(#1)*
 *13.*
*Draw(#10)*



**Figure 4:**
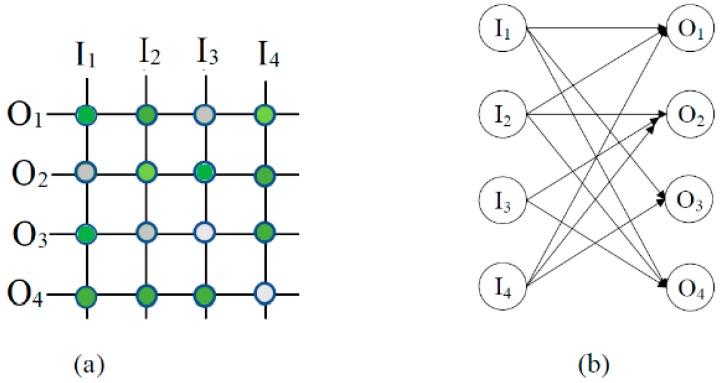
(**a**) A 4 × 4 Defective crossbar and (**b**) the corresponding Bipartite graph.

**Figure 5:**
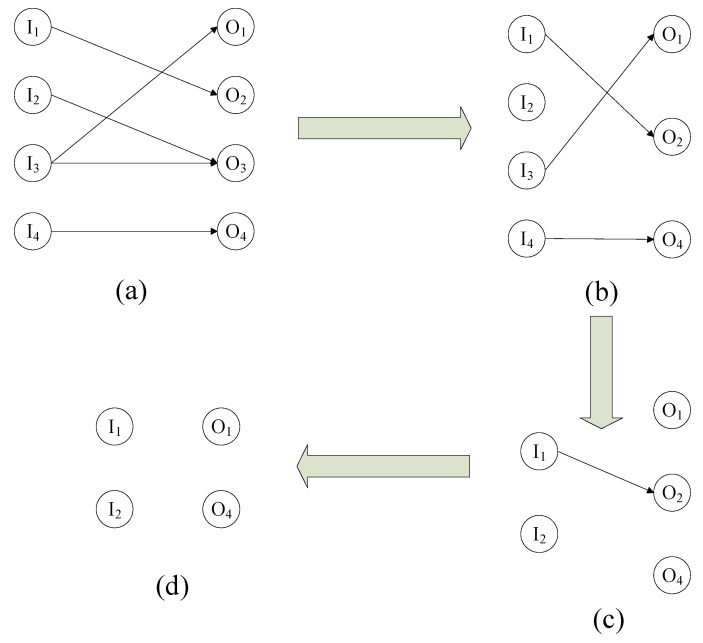
The newly-formed graph after each iteration of the newly-developed defect-tolerant algorithm.

Consider a 4 × 4 defective crossbar as shown in Figure 4a. The defect-free subcrossbar analysis is done based on the assumption that the system has only stuck-open fault as depicted. The bipartite graph and its complement graph are shown in Figure 4b and Figure 5a, respectively. The edges corresponding to the stuck at open faults are eliminated from the complement graph. Figure 5b shows the new graph after the application of the first iteration of the algorithm. This eliminates the node O_3_ and this makes I_2_ as an independent node. In the next iteration nodes I_3_ and I_4_ are eliminated and, hence, O_1_ and O_4_ become independent. The result of this iteration is shown Figure 5c. The next iteration will yield the complete independent set of the complement graph as shown in Figure 5d. In this iteration node O_2_ is eliminated and this result in the independent node I_1_. The complete node set obtained as independent corresponds to the defect-free subcrossbar of the defective bipartite graph considered.

## 4. Results and Discussions

### 4.1. Defect-Free Subcrossbar for Smaller Systems

By applying the newly-developed algorithm to find the defect-free subcrossbar, it is clear that as the defect density increases yield is decreasing. It also indicates that as the original crossbar size increases the yield also slightly increases for the same defect density. The algorithm is applied to three different crossbar sizes with various defect densities.

Initially the method is applied to three different defective crossbar system, which include stuck-at-open faults only. Considered in this study are 4 × 4, 5 × 5, and 6 × 6 crossbars with defect densities of 10% to 80%. The area yield of the defect-free subcrossbar is calculated as the square ratio between the nodes of defect-free subcrossbars with the defective crossbar.

A 4 × 4 crossbar system with a 10% defect has the maximum yield, of about 50%. However, as the defect density increases, yield shows a decreasing trend and it reaches 25% when the defect density reaches 80%. In the case of the 5 × 5 crossbar, the maximum yield is obtained with a defect density of 10% and the minimum is with a defect density of 80%. Similarly, for a 6 × 6 crossbar system, when the defect density is 10% the yield is 22%, while it becomes 11% with a defect density of 80%. Yield is at a minimum in the case of the 6 × 6 crossbar with 80% defects. Thus, even for a smaller sized crossbar system, there is a considerable decrease in yield with the increase in defect density. These results are tabulated in Table 1.

**Figure 6:**
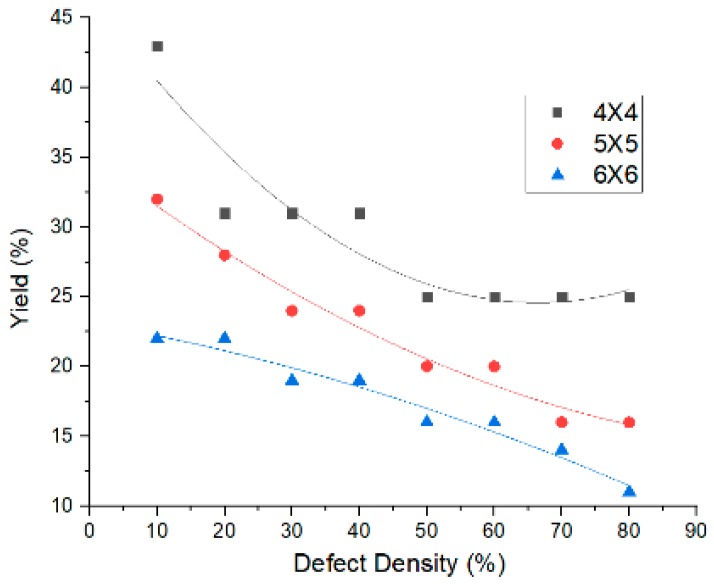
Defect density vs. yield plot for three different crossbar sizes.

A graph is plotted between the percentage yield of the defect-free subcrossbar with the defect density. The graph is plotted for 3 the different crossbar sizes that are analyzed here and is given in Figure 6. From the graph it can be seen that as the defect density and crossbar size increases, the yield of the defect-free subcrossbars decreases.

### 4.2. Defect-Free Subcrossbar for Higher Orders

Higher-order defective crossbar systems of four different sizes are analyzed in terms of defect-free subcrossbar yield through the algorithm developed in this research work. 

#### 4.2.1. 50 × 50 Crossbar

For a 5% defect density, the algorithm gives 27% yield, which is the same as that of the existing algorithms while, for 10% and 15% defect densities, the algorithm gives a yield of 19% and 17%, respectively. These results have a considerable increase in yield from the existing algorithms.

#### 4.2.2. 100 × 100 Crossbar

The yield obtained for a 100 × 100 size crossbar with a defect density of 5, 10, and 15% are 16, 6, and 5%, respectively. Even a 5% increase in the defect density causes a considerable decrease in the defect-free subcrossbar size.

#### 4.2.3. 150 × 150 Crossbar

In all the three different defect densities that are considered, the yield obtained is higher than that of all the four existing algorithms. 

#### 4.2.4. 200 × 200 Crossbar

In this case, also, the yield is considerably high compared to the existing algorithms. 

Due to the NP-complete nature of the maximum independent set finding problem, in some cases the yield is either equal or lower than that of the existing algorithm. But in most of the cases the newly designed fault-tolerant algorithm produces a better area yield.

**Figure 7:**
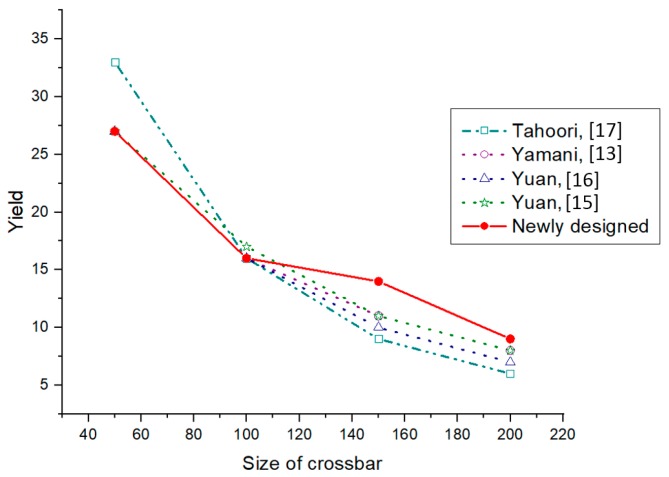
Yield comparison of defect-unaware fault-tolerant methods for a 5% stuck-at-off fault.

**Figure 8:**
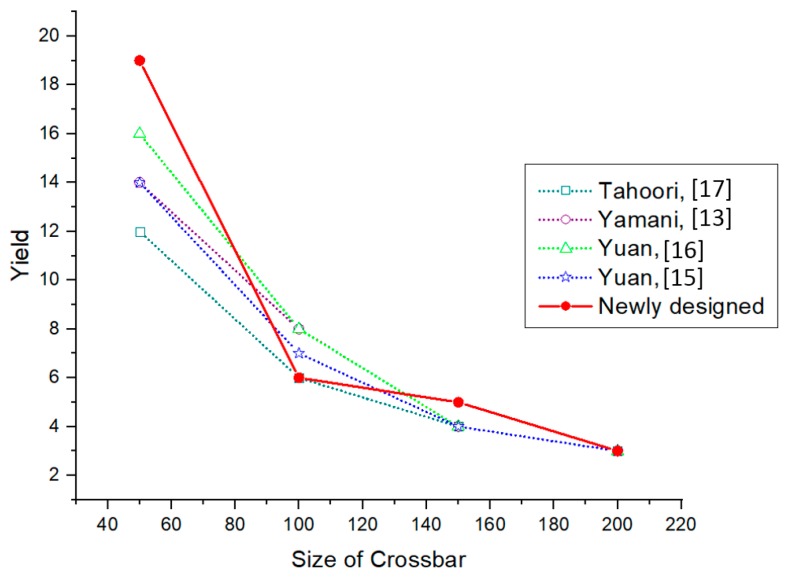
Yield comparison of defect-unaware fault-tolerant methods for a 10% stuck-at-off fault.

**Figure 9:**
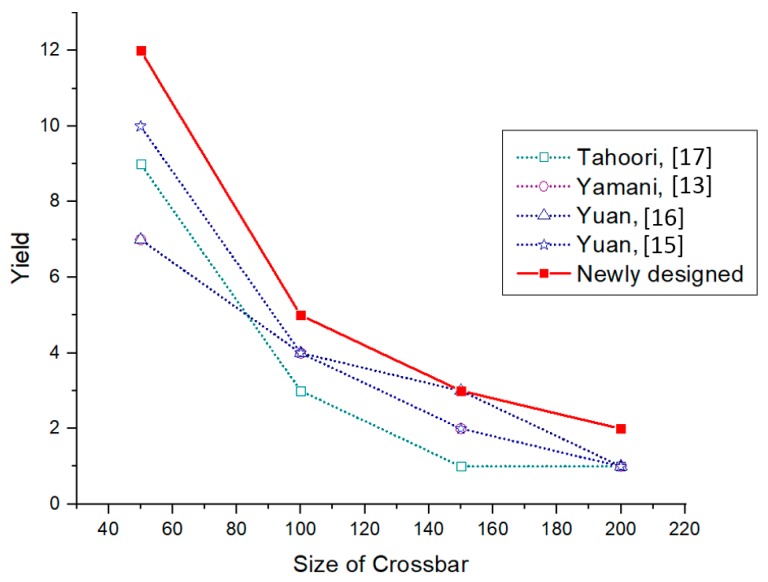
Yield comparison of defect-unaware fault-tolerant methods for a 15% stuck-at-off fault.

Table 2 shows the details of yield comparison for four different sized crossbar architectures (50 × 50, 100 × 100, 150 × 150 & 200 × 200) with three different defect densities. The maximum possible yield from all algorithms in each case is written in bold numbers. Even though only four different crossbar size yield results are presented here, the new algorithm can be applied to any crossbar size with different defect densities. From the defect-free subcrossbar size analysis it can be found that the yield will decrease with the size of the crossbar as well as the defect density. It can be seen that, as the defect density increases, the yield decreases irrespective of the crossbar size. It can also be seen that, for the same defect density, as the crossbar size increases the yield is decreasing. From the yield comparison of all five defect-unaware fault-tolerant algorithms, it can be decided that the newly-designed algorithm has a better yield in most of the cases. A graphical comparison of the yield with respect to the crossbar size is given in Figure 7, Figure 8 and Figure 9 corresponding to defect densities of 5%, 10%, and 15%, respectively.

According to boundary theory, *k* is given that *k*(*n*) ≤ *k* ≤ 2*k*(*n*) with high probability (for sufficiently large n), where *k*(*n*) = log *n*/log (1/(1 − *p*)) and *p* is the defect density [18]. Thus, a very large sized crossbar is insignificant from the point of view of defect-unaware fault-tolerant methods. Nanocrossbar manufacturing is an ongoing field of research and this newly-developed algorithm can be certainly used to achieve a better manufacturing yield.

In the next phase a 4 × 4, a 5 × 5 and a 6 × 6 crossbar are analyzed for 10% stuck-at-open and 10% stuck-at-closed faults. However, it is found that the yield is very low compared to stuck-open faults alone in all three cases. For the 4 × 4 crossbar, the yield is only 31.25% while, for 5 × 5, it is only 16%. When it comes to a 6 × 6 crossbar, the yield is still decreasing and becomes only 8.3%. Hence, even for a 10% defect density for a 6 × 6 crossbar system the yield is too low and it is not cost effective to examine the crossbar systems with higher defect density.

## 5. Conclusions

The newly-developed fault-tolerant system is analyzed for various crossbar sizes with different defect densities. The newly-developed defect-tolerant method is developed based on graph theory by eliminating the all adjacent nodes excluding the minimum degree node. Thus, this method can eliminate more adjacent nodes in each iteration since it is considering more candidate nodes in each pass. This results in a considerable improvement in runtime. It is also clear that, in most of the cases, the newly-developed algorithm gives a better yield compared to that of the existing defect-unaware fault-tolerant logic mapping schemes. 

The fault-tolerant scheme designed here can be applied to defective crossbar system with both stuck-at-on and stuck-at-off fault. However, the presence of stuck-at-on faults leads to a considerable yield loss in the defect-free subcrossbar. The defect-free subcrossbar design is simple but the design flexibility is low compared to defect-aware fault-tolerant methods. The fault-tolerant design can be applied to all types of crossbar architectures irrespective of the type of connecting/switching element.

The fault-tolerant method described here considers only the stuck-at faults. Defect-unaware fault-tolerant methods for other types of faults, like wire defects, transient faults, etc., are yet to be investigated. There exists a possibility of the formation of new defects or the removal of existing defects during the operational lifetime of the crossbar array. The fault tolerance mechanism for these kinds of defects needs to be explored in the future. It can also be found that the maximum biclique finding of a bipartite graph is an NP-complete problem and, hence, there may exist more efficient methods which need to be explored in future.

## Figures and Tables

**Table 1:** Percentage yield analysis for three different sized crossbars with different densities.

Crossbar Size	Defect Density (%)	Yield (%)
4 × 4	10	43
	20	31
	30	31
	40	31
	50	25
	60	25
	70	25
	80	25
5 × 5	10	32
	20	28
	30	24
	40	24
	50	20
	60	20
	70	16
	80	16
6 × 6	10	22
	20	22
	30	19
	40	19
	50	16
	60	19
	70	14
	80	11

**Table 2:** Yield comparison of different defect-unaware fault-tolerant algorithms.

Crossbar Size	Defect Density (%)	Yield				
		Newly-Designed Algorithm	Tahoori [17]	Yamani [13]	Yuan [16]	Yuan [15]
50 × 50	5	27	33	27	27	27
	10	19	12	14	16	14
	15	12	9	7	7	10
100 × 100	5	16	16	16	16	17
	10	6	6	8	8	7
	15	5	3	4	4	4
150 × 150	5	14	9	11	10	11
	10	5	4	4	4	4
	15	3	1	2	3	2
200 × 200	5	9	6	8	7	8
	10	3	3	3	3	3
	15	2	1	1	1	1
